# Role of CSPP-L in recruitment of ciliopathy proteins to centriolar satellites and the ciliary transition zone

**DOI:** 10.1186/2046-2530-1-S1-P36

**Published:** 2012-11-16

**Authors:** S Patzke, J Sternemalm, S Geimer, X Sun, EK Aarnes, T Stokke, LB Pedersen

**Affiliations:** 1 Dept of Radiation Biology, Institute for Cancer Research, OUH - Norwegian Radium Hospital, Norway; 2 Cell Biology/Electron Microscopy, University of Bayreuth, Germany; 3 Dept of Biology, University of Copenhagen, Denmark

## 

We reported earlier that CSPP-L, the larger isoform of the Centrosome/Spindle Pole associated Protein 1, is a ciliary protein required for primary cilia formation in hTERT-RPE1 cells. CSPP-L was found to localize to the basal body and the proximal and distal end of the primary cilium, and to be essential for recruitment of its interaction partner NPHP8 (RPGRIP1L) to the transition zone. Ectopic expression of CSPP-L or depletion of NPHP8 positively regulated cilia elongation, indicating antagonistic activities of these proteins at the primary cilium (Patzke et al., Mol Biol Cell 2010). However, the exact mechanism by which CSPP-L regulates cilia formation and elongation remained elusive. Using immunogold electron microscopy of mouse trachea epithelia cells, we now show that CSPP-L localizes to the plus-end of the ciliary axoneme and the transition fibers of the basal body. CSPP-L could also be detected in extra-centrosomal electron-dense structures - putative centriolar satellites. Indeed, CSPP-L partially co-localized and complexed with centriolar satellite components in hTERT-RPE1 cells. Importantly, CSPP-L depletion decreased formation of PCM-1, CEP290 and EB3 comprising satellites. EB1 and EB3 have recently been shown to be required for ciliogenesis and to complex with these centrosomal proteins in addition to the dynein/dynactin complex (Schroeder et al, J Cell Sci 2011). Interestingly, expression of a dominant negative EB1 (EB1-C-GFP) construct diminished the centrosomal localization of dynactin and CSPP-L. Collectively, these data thus suggest that CSPP-L contributes to ciliogenesis via promoting dynactin-EB complex dependent recruitment of centriolar satellite components to the cilium base.

